# Association of Kidney Stones and Recurrent UTIs: the Chicken and Egg Situation. A Systematic Review of Literature

**DOI:** 10.1007/s11934-022-01103-y

**Published:** 2022-07-25

**Authors:** Francesco Ripa, Amelia Pietropaolo, Emanuele Montanari, B. M. Zeeshan Hameed, Vineet Gauhar, Bhaskar K. Somani

**Affiliations:** 1 Department of Urology, Fondazione IRCCS Ca’ Granda Ospedale Maggiore Policlinico Milano, Milan, Italy; 2 Department of Clinical and Community Science, Università degli Studi di Milano, Milan, Italy; 3 Department of Urology, University Hospital Southampton NHS Trust, Southampton, UK; 4 Department of Urology, Kasturba Medical College Manipal, Manipal Academy of Higher Education, Manipal, Karnataka India; 5 Department of Urology, Ng Teng Fong Hospital, Singapore, Singapore

**Keywords:** UTI, Kidney calculi, Ureteroscopy, Percutaneous nephrolithotomy, Recurrence, Lithotripsy

## Abstract

**Purpose of Review:**

Kidney stone disease (KSD) and recurrent urinary tract infections (rUTI) are frequently concomitant conditions. We conducted a systematic review to determine the association of UTI in patients with KSD and to assess the outcomes of kidney stone treatment in the resolution of rUTI.

**Recent Findings:**

Our systematic review included 17 papers and a strong association between KSD and rUTI was demonstrated by numerous studies. Surgical clearance of kidney stones usually resulted in the resolution of UTI, but discordant data persist regarding recurrence rates after surgery. In vitro studies might unveil the causative role of bacteria in the formation of “metabolic” stones.

**Summary:**

Our SR clearly shows that UTI and KSD are mutually coexisting, and reciprocally causal and such patients should be counselled for proactive intervention by stone removal especially when UTIs are recurrent or additional risk factors are present irrespective of stone composition. To prevent further UTI episodes, if possible, a stone culture must be obtained for an effectively targeted antibiotic treatment regime tailored to bacterial prevalence.

## Introduction

Kidney stones affect approximately 1 in 11 people in the USA, especially obese and diabetic patients [1]. The relationship between kidney stone disease (KSD) and urinary tract infections (UTI) is yet to be clarified where in a chicken and egg dilemma exists as to which is the aetiological factor and which the consequence. UTI have an established etiopathogenetic role in the formation of infection stones (magnesium ammonium phosphate or “struvite” stones, frequently combined with calcium phosphate or calcium carbonate apatite) [2], through the urea-splitting mechanism of urease-producing gram-positive and gram-negative bacterial species such as *Proteus* [3], *Staphylococcus*, *Pseudomonas*, *Providencia*, *Ureaplasma* [4], and *Klebsiella* [5]. However, they comprise 10–15% of urinary stones overall.

Recent investigations are questioning the possible causative role of non-urease-producing bacteria such as Enterobacteriaceae in the onset and growth of non-infected stones, known as “metabolic” stones [6]. Whether the role of UTI in KSD is causative, coincidental or accidental remains to be assessed [7]. With an increased risk of urosepsis in patients with KSD [8, 9] it is imperative to establish if UTI is the primary driving force in stone formation or does bacterial colonisation on stones increase the risk of severe sepsis [8]. Finally, there is a lack of knowledge about the best treatment option that should be offered to patients with kidney stones that may be contributing to recurrent UTIs [10]. Similarly, there is some disagreement about infection-free rates after treatment of urinary stones.

The aim of this study is to investigate the relationship between KSD and UTI and to decrypt evidence for hypothesis which favours that treatment of KSD can provide a resolution to recurrent UTI in this cohort (especially in struvite stones). Finally, we give an overview based on the latest updates in the world literature on hypothesized mechanisms of bacterial impact in stone formation and growth.

## Methods

### Search Strategy

Our systematic review was performed as per the Cochrane guidelines and Preferred Reporting Items for Systematic Reviews and Meta-analyses (PRISMA) checklist [11]. The databases searched included MEDLINE, Excerpta Medica Database (EMBASE), Scopus, Clinicaltrials.gov, Google Scholar, Cochrane Library, and Web of Science with references cross-checked. The search terms included the following: “Kidney stone disease,” “renal stones,” “kidney stones,” “renal calculi,” “urolithiasis,” “nephrolithiasis,” “infected stones,” “struvite,” “urinary tract infections,” “UTI,” “sepsis,” and “infection.” The references of identified studies were examined to find any further potential studies for inclusion. Boolean operators (AND, OR) were employed. The research was limited to English language articles from 2000 to 2022. Areas of interest were:

- Association of UTI and KSD
- Role of KSD treatment in UTI resolution
- Struvite and infection stone

## Results

### Study Selection and Characteristics

The literature search yielded 1900 publications. After excluding reports that were out of the scope of our systematic review, 243 abstracts were evaluated, of which 39 full articles were reviewed for inclusion and 17 articles met the eligibility criteria.

### The Association of UTI and KSD

The concomitant presence of UTI and KSD has been largely demonstrated (Table 1) [12–15]. The first correlation between chronic urinary tract infections and renal aggregations was assessed by Hugosson et al. [16] in 1989 on a cohort of 43 patients with bacteriuria and renal stones. Their aim was to distinguish patients with stone-related infection and bacteriuria vis a vis those with lower urinary tract infection and no KSD, addressing the relationship between cultured microorganisms from ureteric catheterization and KSD. They concluded that by pursuing active stone intervention aimed at eradicating the infection can absolve risk of permanent UTI.

**Table 1:** Studies showing the association of KSD with UTIs

**Author, year**	**Type of study (country)**	**Study population**	**Key findings**
Holmgren et al. (1989) [17]	Retrospective (Sweden)	1325 patients with KSD and UTI	Positive UC in 28% of pts with KSD; pts with UTI more often submitted to surgery
Jan et al. (2008) [18]	Cross-sectional study (Pakistan)	100 patients with UTI and KSD	Frequency of KSD in pts with UTI was 18.98%
Geraghty et al. (2021) [19•]	Retrospective (UK)	819 stone formers vs. 2477 non stone-formers	Pts with KSD had increased risk of UTI (HR 5.73); higher risk of UTI in pts with calcium oxalate (HR 6.36) and urate stones (HR 6.87)
Huang et al. (2012) [20]	Retrospective (Taiwan)	1679 patients < 18 years with KSD and UTI	UTI most common condition associated with KSD (34.1%)
Cetin et al. (2020) [21]	Retrospective (Turkey)	599 patients < 18 years with KSD and UTI	Age at diagnosis, metabolic alterations and size of stone were risk factors for UTI in pts with KSD
Tavichakorntrakool et al. (2012) [8]	Prospective, cohort study (Thailand)	100 patients with KSD and UTI	Prevalence of UTI associated with KSD was 36%
Kitano et al. (2021) [22]	Retrospective (Japan)	286 patients with *S. aureus* UTI	Pts with *S. aureus*-related UTI more likely to have KSD (OR 1.2)
Yilmaz (2012) [23]	Prospective, cohort study (Turkey)	177 patients with KSD and UTI	KSD pts with leukocytosis, pyuria and fever had higher incidence of positive UC
Hsiao et al. (2019) [24]	Retrospective (Taiwan)	662 patients with UTI (549 non-KSD vs. 113 KSD)	Pts with UTI and KSD had increased risk of septic shock (OR 1.80), AKI (OR 1.95) and bacteremia
De Cógáin et al. (2014) [45]	Prospective, cohort study (USA)	125 patients with KSD undergoing PCNL	Non-struvite stones frequently associated with UTI
Xie et al. (2020) [47]	Retrospective (China)	22 stone formers vs. 21 controls	Reduced species diversity and altered microbial profile in KDS pts urine samples

*AKI* acute kidney injury, *KSD* kidney stone disease, *PCNL* percutaneous nephrolithotomy, *pts* patients, *UC* urine culture, *UTI* urinary tract infections

Holmgren et al. reported a 28% incidence of positive urine culture in 1325 adult patients with KSD followed up over a 7-year period, reportedly a high rate as compared to the prevalence of bacteriuria in healthy population [17]. This was considered a high rate compared with the prevalence of bacteriuria found in the healthy population. The frequency of UTI episodes (positive urine culture (UC) per patient) was the highest among patients with *Proteus* infections and in patients with magnesium ammonium phosphate stones (88%), whereas oxalate-containing calculi predominated among patients without infection. In another cohort of 100 patients presenting with urinary symptoms, infection was present in 79% of cases [18]; the most common organisms isolated according to culture report were *E. Coli* (30%), *Proteus* (19%), *Klebsiella* (11%); among infected patients, the frequency of renal stone disease was 18.98% (12.6% in males vs. 6.3% in females).

One of the largest cohorts of kidney stone-formers, compared to a matched cohort of non-stone-formers (819 vs. 2477, respectively), was studied by Geraghty et al. [19•]. This retrospective study, with median follow-up of 19 years, showed that 155 stone formers (18.7%) developed at least one UTI during the study period, compared to 422 (14.1%) of the comparator population. Thus, kidney stone formers showed a significantly increased risk of UTI (HR 5.73; 95% CI 4.55–7.21, *p* < 0.001). Of those 155 stone formers who developed a UTI, 63 had at least one stone recurrence, once again revealing the tight link between these conditions. The association of UTI with stone composition was assessed as well, demonstrating significantly higher risk of UTI in patients presenting with calcium oxalate stones (HR 6.36; 95% CI 4.82–8.40, *p* < 0.001) and urate stones (HR 6.87; 95% CI 2.82–16.72, *p* < 0.001), compared to other stone compositions.

Similarly, a nationwide study from Taiwan stated that UTI was the most common associated morbidity among 1679 pediatric subjects with newly diagnosed urolithiasis, in 34.1% of all subjects [20]. In a retrospective follow-up study of 599 pediatric patients with nephrolithiasis under 2 years old at diagnosis, presence of a metabolic risk factor and size of stone > 5.3 mm were assessed as significant risk factors for the onset of single and recurrent UTI in children with nephrolithiasis [21].

In a prospective study analyzing 100 stone former patients admitted for elective kidney stone removal from Thailand [8], the prevalence of UTI associated with nephrolithiasis was up to 36%. Both catheterized urine samples and stone matrices cultures were performed in this study, obtaining 45 different bacterial isolates. Among these, the most common species found in urine samples were *Escherichia coli*, *Enterococcus* spp. and *Klebsiella/Enterobacter* spp., whereas those found in stone matrices were *E. coli*, *P. mirabilis* and *Klebsiella* spp. This addressed the problem of understanding whether these bacteria might induce the formation of the so-called infection-induced stones or if they were just a subsequent finding of a secondary infection “stones with subsequent infection.” For this reason, authors selectively obtained cultures from the stone “nidus” or core, searching for *causative* bacteria, and from the peripheral part of the calculus, looking for bacterial strains that might have colonized the pre-existent stone during subsequent episodes of UTI. Interestingly, the types of bacteria found in the stone nidus were almost identical to those found in the stone periphery, and a great concordance rate between microbiological isolates from urine culture collected from catheterized samples and stone matrices was found (*r* = 0.860, *p* < 0.001), with the authors concluding that the bacteria were likely causative for stone formation and growth rather than stone colonization. Bacteria isolated from both urine and stone matrices had multidrug resistance: in catheterized urine samples, 19 (70%) had antimicrobial resistance; in stone matrices, 24 (62%). Interestingly, 69% (25 of 36) of isolated microorganisms were non-urea-splitting bacteria vs. 31% (11 of 36) of urea-splitting bacteria. Fifteen “infection-induced stones” and 85 “metabolic stones” were found. This proportion remained when compared among 36 stone formers with positive bacterial isolates. Thus, recurrent UTIs were associated with almost all kidney stone compositions.

Kitano et al. [22] evaluated 286 patients with *S aureus* bacteriuria who presented with UTI and without UTI over 7 years, and reported a significant association of indwelling catheters, renal stones and presence of hydronephrosis in the former (*p* = 0.002, 0.04, < 0.001, respectively), confirming a positive association between KSD and UTI.

Yilmaz et al. [23] analyzed a cohort of 192 patients with urolithiasis presenting to emergency department; 27 patients (15.3%) had a positive urine culture whereas the remaining 150 patients (84.7%) had a negative urine culture. Using ROC analysis, authors found that pyuria (over 10 WBCs per HPF), fever over 37.9 °C, and leukocytosis over 11,300/mm^3^ were the best predictors of a positive urine culture among patients presenting with urolithiasis, concluding that urinary stones may increase the risk of UTI. They also demonstrated that the gold standard for the diagnosis of urinary tract infection in patients with KSD was urine culture.

A retrospective observational study was conducted in Taiwan enrolling 662 consecutive patients hospitalized for UTI [24]. All patients underwent radiological imaging to detect the presence of urolithiasis and eventual urinary tract obstruction. A comparison between those with and without stones was performed. The prevalence of male sex (40.7 vs. 27.0%, *p* = 0.003), bacteremia (57.5 vs. 40.3%, *p* = 0.001), uroseptic shock (26.5 vs. 14.0%, *p* = 0.001), acute kidney injury (40.7 vs. 25.1%, *p* = 0.001), and *Proteus* spp. isolates (10.6 vs. 2.6%, *p* < 0.001) was higher, while *E. coli* isolates (61.1 vs. 75.8%, *p* = 0.002) was lower in patients with urolithiasis compared to those without. A higher prevalence of urolithiasis in UTI patients than in the general population was assessed. Moreover, the presence of urolithiasis was associated with worse clinical outcomes in UTI patients.

### Eradication of Stones Might Lead to UTI Resolution

The strong association between KSD and UTI progressively led authors to consider the clinical implications, with infections harbored either in patients with recurrent and relapsing UTI or in patients presenting with urinary tract stones demanding treatment (Table 2). Here the “chicken and egg” dilemma is reversed, raising the hypothesis that the eradication of kidney stones through surgical intervention might result in substantial reduction in bacterial burden reducing recurrent UTIs and or bacteriuria.

**Table 2:** Outcomes of intervention and resolution of UTI

**Author, year**	**Study, (country)**	**Population**	**Age (median); males; females**	**Stone composition**	**Pre-operative UC + **	**Pre-operative recurrent UTI**	**Procedures (n, type)**	**SFR**	**UAS**	**Length of stay**	**Complications, n, (type)**	**Follow up (FU)**	**Outcomes**
Hugosson et al. (1989) [16]	Retrospective (Sweden)	43 patients with bacteriuria and renal stones	58 (22–83); 14; 29	14 struvite/carbonate apatite; 9 CaOx/CaP	*E. coli* 18; *Proteus* 11; *Klebsiella* 2; *Enterobacter cloacae* 1; *Pseudomonas* 1; *Gardnerella vaginalis* 1; *S. saprophiticus* 1; *S. epidermidis* 2; *E. faecalis* 3; *Streptococcus* 1; Multiple 2	Bacteriuria: 15 for > 10 years; 11 for 5–10 years; 15 for 2–5 years; 2 for < 2 years	23 (15 PCNL; 4 open nephrolithotomy; 1 TUR extraction; 2 nephrectomies; 1 upper pole resection)	NA	NA	NA	NA	23 months (10–48)	87% free from infection at FU; 13% persistent bacteriuria after surgery (residual fragments)
Oliver et al. (2017) [25•]	Prospective, cohort study (UK)	103 patients with rUTI or pre-operative UC + who underwent URS	60 (21–89); 37; 66	63 CaOx (61.2%); 17 struvite (16.5%);	81 (78.6%) UC + ; *E. coli* 31 (38.3%); Enterococcus spp 10 (2.3%); Coliforms 8 (9.8%); Yeast 7 (8.6%); *Proteus* 4 (4.9%); *Pseudomonas* 3 (3.7%); *Staphylococcus* 2 (2.5%); *Klebsiella* 2 (2.5%); multiple 14 (17.3%)	22 (21.4%)	115 URS (94 unilateral; 9 bilateral; 12 2–staged)	96%	42 (41%)	1.4 ± 3.8 days	13 (12.6%) UTI (*n* = 3), sepsis (*n* = 7), stent pain (*n* = 3)	3, 6, 12 months	3 m: SFR 96%, IFR 88%; 6 m: SFR 90%, IFR 86%; 12 m: SFR 82%, IFR 71% (*p* < 0.001)
Agarwal et al. (2020) [26•]	Retrospective (USA)	46 patients with rUTI who underwent a procedure for urolithiasis	63.7 (49.2–73.4); 4; 42	CaOx monohydrate 15; CaOx dihydrate 3; apatite 11; brushite 2; uric acid 5; mixed 14	*E. coli* 17 (37%), Enterococcus spp 8 (17%), *Klebsiella* 6 (13%)	≥ 3 UTI in 12 months, with symptoms and UC +	43% URS; 57% PCNL ± URS	63% URS; 65% PCNL;	NA	NA	3 (2 SIRS/sepsis, 1 readmission)	2.9 years (IQR 2.0–4.3)	89% rUTI-free; average 3.1 UTI before surgery vs 0.5 after surgery (*p* < 0.001); residual stone associated with rUTI (*p* = 0.046)
Omar et al. (2015) [27•]	Retrospective (USA)	120 patients with rUTI who underwent surgical stone extraction	59.5 ± 18.1; 38; 82	CaOx 29; CaP 49; struvite 6; uric acid 7; mixed 2	*E. coli* 62; *Klebsiella* 24; *Proteus* 13; *Pseudomonas* 17; *Citrobacter* 6; *Enterococcus* 26	3 or more UTIs per year, or 2 or more in 6 months	URS 8 (6.6%); PCNL 73 (60.8%); ESWL 39 (32.5%)	NA	NA	2.95 ± 3.9 days	16 (13.3%) SIRS	14 ± 3 months	58 pts (48%) infection-free after surgery 62 pts (52%) rUTI

*CaOx* calcium oxalate, *CaP* calcium phosphate, *ESWL* extracorporeal shock wave lithotripsy, *IFR* infection-free rate, *NA* not available, *PCNL* percutaneous nephrolithotomy, *rUTI* recurrent urinary tract infections, *SIRS* systemic inflammatory response syndrome, *SFR* stone-free rate, *UC* + positive urine culture, *TUR* trans-urethral resection, *URS* ureteroscopy, *UAS* ureteral access sheath

A prospective, cohort-study involving 103 patients who underwent URS for stone treatment with a history of recurrent UTIs or a positive pre-operative urine culture was conducted between March 2012 and July 2016 in the UK [25•]. Mean stone size was 16.4 mm, and 81 patients (78.6%) had preoperative positive urine culture, whereas 22 (21.4%) had recurrent UTI. Single organisms were present in 67 (82.7%) cases, multiple bacteria in 14 (17.3%). Coliforms (*n* = 51, 63%) and urease-producing bacteria (26%, *n* = 21) were the most common. A total of 115 procedures were performed, with a stone-free rate (SFR) of 96%. Stone-free and infection-free rates (IFR) were assessed at preset endpoints of 3, 6 and 12 months. A strong association between KSD and UTIs was demonstrated, as clearance of stones led to resolution of UTI in the majority of cases. At 3 months, SFR was 96% and IFR was 88%; at 6 months (*n* = 90), the SFR and IFR were 90% and 86%; at 12 months (*n* = 82), the SFR and IFR were 82% and 71%, respectively (*p* < 0.001) a strong indicator that as SFR increased IFR dropped. Furthermore, 8/10 (80%) patients with stone recurrence also had a recurrent episode of UTI proving that KSD and UTI are directly coexisting. On the other hand, 75% of stone-free patients were UTI free at 12 months (*p* < 0.001).

In order to investigate the role of surgical procedures for non-obstructive urolithiasis in the relief from recurrent UTIs, Agarwal et al. [26•] examined a retrospective cohort of 46 patients with recurrent UTI (proved by presence of symptoms and a positive culture) submitted to either URS (43%) or PCNL (57%) between 2009 and 2016. Mean stone size was 20 mm (IQR 14–35) and median follow-up was 2.9 years (IQR 2.0–4.3). *Escherichia coli*, *Enterococcus* spp., and *Klebsiella pneumoniae* were causative bacteria in 17 (37%), 8 (17%), and 6 (13%) patients respectively. Patients were treated with preoperative antibiotics. SFR was 63% for URS vs. 65% for PCNL. After surgery, 22% required a second stage operation, with median residual fragment size of 3 mm (IQR 2–6). Those with recurrent UTI postoperatively were compared to those without. Results showed that 68% of patients were able to discontinue pre-operative prophylaxis after surgery. Fifty-two percent of patients had a single episode of UTI > 30 days after surgery, at a median time of 12.3 months (IQR 5.2–27.8), but 89% of the cohort was free of recurrent UTIs postoperatively, enhancing the effective role of surgical clearance of kidney stones, even though the effect of antibiotics given at the time of or before stone removal cannot be ignored. 80% in the UTI group had recurrent UTIs (rUTIs) caused by the same pathogen identified preoperatively. An average of 3.1 UTIs in the year prior to surgical intervention vs. 0.5 UTI in the following year was assessed (*p* < 0.001). Interestingly, presence of residual stone was the only statistically significant difference between rUTI patients compared to those without (*p* = 0.046), with no significant association with stone size, composition, type of procedure, and stone culture with postoperative rUTI.

Omar et al. [27] in 2015 retrospectively analyzed a cohort of 120 patients with rUTIs who underwent surgical stone extraction. Recurrent UTIs were defined as 3 or more UTIs per year, or 2 or more in preceding 6 months. Group 1 had no evidence of recurrent infection 1 year after stone removal, whereas recurrent infection developed in group 2. Median follow-up was 14 ± 3 months; mean stone size was 14.1 ± 8.4 mm vs. 15.2 ± 9.8 mm (group 1 vs. 2, respectively). URS was performed in 3 (5%) vs. 5 (8%) cases; PCNL in 39 (67%) vs. 34 (55%) cases and ESWL in 16 (28%) vs. 3 (37%) cases (group 1 vs. 2, respectively). Infection with *E. coli* only was associated with successful clearance of infection (*p* < 0.01). On the contrary, infection with *Enterococcus* was associated with failed clearance rate (*p* = 0.04). The infection-free rate after surgery was much lower than Agarwal et al.: only 58 patients (48%) were rendered infection-free with stone eradication, while 62 patients (52%) continued to have infections, with a mean time to the first recurrent UTI of 12 ± 2 months. Stone composition and type of stone removal procedure were not associated with recurrent UTIs. Among post-operative rUTI, 82% continued to have infections with the same preoperative organism, vs. 18% who had a change in bacterial species. After univariate and multivariate analysis, risk factors such as black American ethnicity, hypertension, and male gender with DM were associated with unsuccessful clearance of infections, suggesting that in these patients, stone extraction may not completely eradicate infection risk. Again, it was raised the issue that the antibiotic course administered before or after surgery (either prophylaxis or treatment) might alter the real effect of the surgical procedure in the resolution of UTI.

This concern was clearly expressed by the group of Zhao and Zeng [28], who highlighted the need to specify the range of preoperative bacterial strains and their proportion of multidrug resistance, the rate of infection clearance in the group of patients with residual stones and the diagnosis of temporal succession between stones and recurrent UTIs, in order to correctly understand the causative link between nephrolithiasis and UTI.

### Bacterial Role in Stone Formation and Growth

Struvite (magnesium ammonium phosphate, MAP) stones have been extensively characterized [5, 29–32]. They are often found incidentally in examinations for abdominal or back pain, recurrent UTI or hematuria, and they frequently form large, branched stones known as staghorn calculi. They are less common in males than females (3.8% vs. 11.0%) and their incidence has been decreasing from 4.9 to 3.3% in males and 13.5 to 9.2% in females over the past decades [33]. Known risk factors for struvite stones onset are female gender, extreme ages, congenital urinary tract malformations, urinary obstruction or diversion, neurogenic bladder, indwelling catheters, distal renal tubular acidosis, medullary sponge kidney, and diabetes mellitus [5].

Its etiopathogenesis is based on urease enzyme-producing bacteria, that could belong either to gram negative or gram positive species [5]. Among these, the most common bacteria involved in struvite formation are *Proteus*, *Staphylococcus*, *Pseudomonas*, *Providencia*, *Klebsiella*, and *Serratia*. Urease from bacteria splits urea into ammonia and carbon dioxide. Ammonia reacts with water to become ammonium and hydroxide ions, which creates an alkaline environment where ammonium reacts with magnesium, phosphate, and water to form MAP stones [34].

If left untreated, infected stones might affect patients with a great burden of morbidity and mortality, mainly to attribute to chronic renal failure or sepsis. Therefore, the aim of active treatment should be the complete eradication of calculi, which is associated with high success rates of stone clearance, low rates of recurrence, and consequent morbidity and mortality. Treatment strategies include antibiotic therapy (even though no guidelines on fixed regimens exist [35]), while PCNL is considered the gold-standard approach for staghorn calculi. Adjunctive options such as urease inhibitors and urine acidification agents are not widely used.

Additional molecular mechanisms have been studied to comprehend crystal aggregation and stone growth. It has been hypothesized that bacterial polysaccharides of the genus *Proteus*, macromolecules that contain negatively charged residues and are able to bind Ca(2 +) and Mg(2 +), might lead to the accumulation of these ions around bacterial cells and accelerate the crystallization process [36, 37]. An in vitro model was used to study intracellular growth and crystallization in the presence of bacterial strains of *P. mirabilis*, *Klebsiella pneumoniae*, and *Escherichia coli* [38]. *P. mirabilis* had the ability to form crystals inside epithelial host cells, protected from antibiotics and from the immune system, leading to persistent and recurrent infections.

*P. mirabilis* is also a well-known cause of catheter-associated urinary tract infections (CAUTIs) in patients with long-term indwelling urinary catheters, due to its ability to form crystalline biofilms that allow its colonies to survive in hostile conditions. This is a matter of concern because antimicrobial resistance (AMR) of biofilm-associated bacteria has been demonstrated to be 10–1,000 times higher compared to their “free” counterparts [39]. The most important virulence factors linked to *Proteus* ability to form biofilms are its swarming motility, fimbriae, urease production, capsule polysaccharide, and efflux pumps [40]. In 2017, Hobbs et al. [41] produced an in vitro model to represent the urinary tract and study the biofilm-induced stone formation. Authors demonstrated the upstream migration of microbes from bladder to kidney, biofilm growth, and stone formation in the experimental kidney. Crystals that formed in the system resembled clinically removed struvite stones in structure and composition.

Nanobacteria may also act as a nucleus for the initiation of the renal stones [42–44]. The question whether non-struvite infected calculi might result from a nidus of bacteria-induced crystallization that becomes then secondarily infected was addressed by De Cógáin et al. [45] on a cohort of 125 patients undergoing PCNL between 2009 and 2012, with subsequent stone culture and metabolic evaluation. Authors stated that non-struvite infected stones were mainly caused by *E. coli* and *Enterococcus* infections; they hypothesize that organisms cultured from stones, with or without a clinically related UTI, could initially cause kidney cell injury and inflammation, which could in turn boost crystal retention and stone formation [46].

Calcium stone was furtherly investigated by a retrospective study conducted by Xie et al. [47] on a group of 22 kidney stone formers and 21 age-matched healthy volunteers, analyzing the bacterial profile from bladder and upper tract urine collected by ureteral catheterization. The stone composition comprised of 18 calcium oxalate stones, 3 calcium oxalate + calcium phosphate stones, and 1 calcium oxalate + uric acid stone. Nephrolithiasis patients had significant lower species diversity in urine. Additionally, a similarity of overall bacterial composition between bladder and renal pelvis urine in kidney stone patients was found. Several functional pathways and bacteria were associated with inflammation in the urinary tract of kidney stone patients. This led the authors to hypothesize that bacteria could adhere to crystals and promote their growth and aggregation, alter urine supersaturation via production of the enzyme citrate lyase, and influence the formation of calcium-based kidney stones via the modulation of inflammatory process and the release of proinflammatory proteins which form the stone matrix inner core.

Moreover, patients believed to have non-infection urolithiasis often present with stone cultures indicating infection by non-urealytic bacteria, such as *Escherichia coli*. In 2015, a prospective cohort study on pediatric patients with kidney stones, followed by in vitro and in vivo (murine) study on the interaction between Enterobacteriaceae and calcium oxalate stones was published [48]. These patients, of a median age of 14.4 years, were submitted to kidney stone removal procedure between 2013 and 2014 (3 URS, 60% vs. 2 PCNL, 40%). Forty percent of them had a previous reported history of recurrent UTI, but none of them was diagnosed with a UTI during or 30 days prior to the stone removal procedure. Pediatric kidney stones and urine were collected and both cultured and sequenced, using Enhanced Quantitative Urine Culture (EQUC) technique and DNA extraction and sequencing. Bacterial DNA and live bacteria were detected in kidney stones, upper tract urine and bladder urine. When detected in bladder urine, the taxa were similar to those observed in the stones. In vitro, CaOx monohydrate and dihydrate crystals were mixed with colony forming units (CFU) of uropathogenic *E. coli* (UPEC); then, the area of crystal was quantified. Interestingly, UPEC aggregate on and around CaOx monohydrate crystals in significantly greater numbers compared to controls, suggesting a causative role in crystal aggregation. Finally, in the in vivo study, CaOx deposits were induced in mice by injecting sodium glyoxalate intraperitoneally, while experimental pyelonephritis was induced by inoculating UPEC. Renal CaOx deposits increased the bacterial burden following UPEC inoculation; at the same time, UPEC inoculation resulted in increased CaOx deposition through an increased expression of stone matrix proteins.

In 2013, Chutipongtanate et al. [49] used spectrophotometric oxalate-depletion assays and CaOx crystal aggregation–sedimentation studies to assess CaOx crystal growth and aggregation, respectively. *E. coli*, *K. pneumoniae*, *S. aureus*, and *S. pneumoniae* promoted CaOx crystal growth and aggregation in a dose-dependent manner. Authors hypothesized that the anionic nature of bacteria or their secretory products might attract free Ca2 + from renal tubular fluid, resulting in promotion of CaOx crystal growth. Additionally, intact viable bacteria interacting on the CaOx surface might act as linkers or adhesive molecules, enhancing the aggregation process.

Finally, *E. coli* has been demonstrated to enhance the expression of osteopontin and mucosal damage in renal tubular cells of a rabbit model [50] that may allow further crystal retention and nucleation resulting in stone formation.

## Discussion

### Clinical Relevance of the Study

This is the first systematic review of all literature findings and recent major evidence on the relationship between kidney stone disease and urinary tract infections. Our study supports the evidence that patients suffering from KSD often present with recurrent or concomitant urinary tract infections. We also see that surgical clearance of kidney stones usually results in the resolution of UTI, although recurrence may happen especially in non-stone-free patients and those presenting with additional risk factors. Finally, emerging evidence clarified with in vitro studies the possible causative role of bacterial strains in the formation and growth of stones previously classified as exclusively “metabolic” stones. This might shed light on the etiopathogenesis of KSD and provide insights for its prevention and treatment.

With regard to UTI in patients presenting with KSD, the concomitant presence of these conditions has been widely assessed [8, 17, 19•, 20], even though percentages vary from 18,7% [19•] to 36% [8]. This might be explained mainly by the different sample cultures and techniques (bladder urine [8, 17, 47] vs. renal pelvis urine from ureteric catheterization [16, 47] vs. stone matrices cultures [8]) and by the different definitions of UTI considered (bacteriuria/positive UC vs. concomitant urinary symptoms or fever [26•]). Moreover, inconsistent results appeared when comparing the risk of UTI according to different stone composition, showing unexpectedly higher risk in calcium oxalate and uric acid stones [19•] or calcium oxalate mixed with phosphate, magnesium ammonium phosphate, and uric acid stones [8], once again proving that colonized urine or stone samples are not exclusively related to struvite and infection stones but may involve almost all chemical stone composition.

According to recent literature findings, treatment of kidney stones might reduce the risk of infections in patients with a reported history of recurrent UTI after the surgical removal. Interestingly, this association was proved significant irrespective of the type of surgical procedure performed, should it be URS [25•], URS ± PCNL ([26•], URS vs. PCNL vs. ESWL [27], even though Omar et al. excluded patients with residual stones from analysis, while Agarwal et al. considered the presence of residual stone as the only significant difference between patients with rUTI compared to those without. The link between ESWL and recurrent infections may be secondary to residual fragments after shock wave therapy that might represent a persistent infective focus. Again, percentages of infection clearance after stone removal range from 75% at 12 months follow-up [25•] to 89% of patients with recurrent UTIs cured after stone extraction [26•], to 48% of infection-free rate after surgery [27]. These discordant data might be explained by the likely effect of pre-operative antibiotic treatment administered to patients presenting with positive urine culture. Therefore, it is difficult to quantify the beneficial effect of stone removal on the prevention of post-operative UTI. However, it has been assessed that UTI recurrence was associated with stone recurrence [25•, 26•], risk factors such as indwelling or intermittent catheterization, diabetes mellitus and contralateral stones [25•], black American ethnicity, hypertension and *E. coli* infections [27]. Finally, microbiological research is focusing on highlighting through in vitro and in vivo studies the potential bacterial mechanisms that might be responsible for the primary onset of crystal aggregations in kidneys. These might comprehend altered species diversity, urine supersaturation [47], as well as bacterial capacity to adhere to the uroepithelial mucosa, to CaOx aggregates [27]. Molecular pathways related to inflammation might trigger stone onset that might explain the higher infection-rate and sepsis-rate associated with patients with IBD and concomitant urolithiasis compared to non-IBD patients [51].

## Limitations and Future Research

This systematic review gives an overview on the most relevant findings on the association between UTI and KSD, highlighting the pathogenetic mechanisms of struvite stone formation and summarizing the most recent hypothesis on the involvement of non-urealytic Enterobacteriaceae in the onset of metabolic stones through in vitro and in vivo settings. The heterogeneity of the selected studies precludes a meta-analysis. Moreover, the discordance on selection criteria of patients, on the microbiological samples analyzed, as well as the different definitions of UTI and recurrent UTIs add several limitations and bias. Finally, the administration of antibiotic treatments in the pre-operative setting is hugely influenced by clinical practice and driven by local microbiological scenarios and sensitivity patterns, which prevent studies from being reliable and comparable.

Future work should focus on enhanced techniques of bacterial cultures and should analyze the primitive molecular mechanism underlying the crystallization of organic and inorganic components in urine in order to finally solve the “chicken and egg” dilemma. Perhaps, a real cost and quality of life (QoL) analysis of treatment vs. surveillance in these patients should also be considered [52, 53].

## Conclusion

Our SR clearly shows that UTI and KSD are mutually coexisting, and reciprocally causal and such patients should be counselled for proactive intervention by stone removal especially when UTIs are recurrent or additional risk factors are present irrespective of stone composition. To prevent further UTI episodes, if possible, a stone culture must be obtained for an effectively targeted antibiotic treatment regime tailored to bacterial prevalence.
